# Supplementary material for: Pyruvate Dehydrogenase Kinase 1 inhibition mediated oxidative phosphorylation enhancement in cartilage promotes osteoarthritis progression
Source: BMC Musculoskelet Disord. 2023 Jul 20;24:597. doi: 10.1186/s12891-023-06585-6 (PMC10357736; doi:10.1186/s12891-023-06585-6)
Supplement: Supplementary file 1 — Additional file 1. [file 12891_2023_6585_MOESM1_ESM.zip › MF.Gsea.1653627316242/GOMF_LIGASE_ACTIVITY.html]

Details for gene set GOMF\_LIGASE\_ACTIVITY[GSEA]

|  || Dataset | 55457\_collapsed\_to\_symbols.cls.cls #OA\_versus\_normal.cls.cls #OA\_versus\_normal\_repos |
| Phenotype | cls.cls#OA\_versus\_normal\_repos |
| Upregulated in class | OA |
| GeneSet | GOMF\_LIGASE\_ACTIVITY |
| Enrichment Score (ES) | 0.23967247 |
| Normalized Enrichment Score (NES) | 1.1664549 |
| Nominal p-value | 0.14480874 |
| FDR q-value | 0.6098425 |
| FWER p-Value | 1.0 |
Table: GSEA Results Summary

  

Fig 1: Enrichment plot: GOMF\_LIGASE\_ACTIVITY      
 Profile of the Running ES Score & Positions of GeneSet Members on the Rank Ordered List

  

| SYMBOL | TITLE | RANK IN GENE LIST | RANK METRIC SCORE | RUNNING ES | CORE ENRICHMENT || 1 | PCCB | propionyl-CoA carboxylase subunit beta [Source:HGNC Symbol;Acc:HGNC:8654] | 29 | 1.057 | 0.0243 | Yes |
| 2 | DALRD3 | DALR anticodon binding domain containing 3 [Source:HGNC Symbol;Acc:HGNC:25536] | 71 | 0.957 | 0.0451 | Yes |
| 3 | RTCB | "RNA 2',3'-cyclic phosphate and 5'-OH ligase [Source:HGNC Symbol;Acc:HGNC:26935]" | 78 | 0.947 | 0.0685 | Yes |
| 4 | GSS | glutathione synthetase [Source:HGNC Symbol;Acc:HGNC:4624] | 502 | 0.658 | 0.0519 | Yes |
| 5 | SARS1 | seryl-tRNA synthetase 1 [Source:HGNC Symbol;Acc:HGNC:10537] | 516 | 0.654 | 0.0673 | Yes |
| 6 | RTCA | RNA 3'-terminal phosphate cyclase [Source:HGNC Symbol;Acc:HGNC:17981] | 519 | 0.653 | 0.0836 | Yes |
| 7 | ATP5PD | ATP synthase peripheral stalk subunit d [Source:HGNC Symbol;Acc:HGNC:845] | 564 | 0.635 | 0.0961 | Yes |
| 8 | EPRS1 | glutamyl-prolyl-tRNA synthetase 1 [Source:HGNC Symbol;Acc:HGNC:3418] | 581 | 0.629 | 0.1106 | Yes |
| 9 | XRCC4 | X-ray repair cross complementing 4 [Source:HGNC Symbol;Acc:HGNC:12831] | 602 | 0.622 | 0.1247 | Yes |
| 10 | PPCS | phosphopantothenoylcysteine synthetase [Source:HGNC Symbol;Acc:HGNC:25686] | 716 | 0.589 | 0.1307 | Yes |
| 11 | TTLL1 | tubulin tyrosine ligase like 1 [Source:HGNC Symbol;Acc:HGNC:1312] | 790 | 0.572 | 0.1393 | Yes |
| 12 | ATP5PO | ATP synthase peripheral stalk subunit OSCP [Source:HGNC Symbol;Acc:HGNC:850] | 840 | 0.558 | 0.1495 | Yes |
| 13 | SLC27A6 | solute carrier family 27 member 6 [Source:HGNC Symbol;Acc:HGNC:11000] | 1182 | 0.480 | 0.1349 | Yes |
| 14 | HARS2 | "histidyl-tRNA synthetase 2, mitochondrial [Source:HGNC Symbol;Acc:HGNC:4817]" | 1195 | 0.478 | 0.1459 | Yes |
| 15 | QRSL1 | glutaminyl-tRNA amidotransferase subunit QRSL1 [Source:HGNC Symbol;Acc:HGNC:21020] | 1250 | 0.467 | 0.1534 | Yes |
| 16 | NAE1 | NEDD8 activating enzyme E1 subunit 1 [Source:HGNC Symbol;Acc:HGNC:621] | 1276 | 0.463 | 0.1631 | Yes |
| 17 | ATP5PB | ATP synthase peripheral stalk-membrane subunit b [Source:HGNC Symbol;Acc:HGNC:840] | 1293 | 0.459 | 0.1734 | Yes |
| 18 | ATP5MF | ATP synthase membrane subunit f [Source:HGNC Symbol;Acc:HGNC:848] | 1330 | 0.453 | 0.1820 | Yes |
| 19 | SUCLG2 | succinate-CoA ligase GDP-forming subunit beta [Source:HGNC Symbol;Acc:HGNC:11450] | 1472 | 0.432 | 0.1818 | Yes |
| 20 | DARS1 | aspartyl-tRNA synthetase 1 [Source:HGNC Symbol;Acc:HGNC:2678] | 1558 | 0.419 | 0.1857 | Yes |
| 21 | UCHL1 | ubiquitin C-terminal hydrolase L1 [Source:HGNC Symbol;Acc:HGNC:12513] | 1585 | 0.416 | 0.1941 | Yes |
| 22 | IARS2 | "isoleucyl-tRNA synthetase 2, mitochondrial [Source:HGNC Symbol;Acc:HGNC:29685]" | 1685 | 0.401 | 0.1964 | Yes |
| 23 | FARS2 | "phenylalanyl-tRNA synthetase 2, mitochondrial [Source:HGNC Symbol;Acc:HGNC:21062]" | 1686 | 0.401 | 0.2065 | Yes |
| 24 | ACSM3 | acyl-CoA synthetase medium chain family member 3 [Source:HGNC Symbol;Acc:HGNC:10522] | 1835 | 0.380 | 0.2044 | Yes |
| 25 | QARS1 | glutaminyl-tRNA synthetase 1 [Source:HGNC Symbol;Acc:HGNC:9751] | 1913 | 0.371 | 0.2077 | Yes |
| 26 | MTHFS | methenyltetrahydrofolate synthetase [Source:HGNC Symbol;Acc:HGNC:7437] | 2155 | 0.343 | 0.1975 | Yes |
| 27 | CPS1 | carbamoyl-phosphate synthase 1 [Source:HGNC Symbol;Acc:HGNC:2323] | 2178 | 0.341 | 0.2043 | Yes |
| 28 | ATP5F1C | ATP synthase F1 subunit gamma [Source:HGNC Symbol;Acc:HGNC:833] | 2190 | 0.339 | 0.2120 | Yes |
| 29 | SUCLG1 | succinate-CoA ligase GDP/ADP-forming subunit alpha [Source:HGNC Symbol;Acc:HGNC:11449] | 2269 | 0.330 | 0.2142 | Yes |
| 30 | ASS1 | argininosuccinate synthase 1 [Source:HGNC Symbol;Acc:HGNC:758] | 2466 | 0.307 | 0.2065 | Yes |
| 31 | GATB | glutamyl-tRNA amidotransferase subunit B [Source:HGNC Symbol;Acc:HGNC:8849] | 2515 | 0.302 | 0.2104 | Yes |
| 32 | ACSBG1 | acyl-CoA synthetase bubblegum family member 1 [Source:HGNC Symbol;Acc:HGNC:29567] | 2520 | 0.301 | 0.2176 | Yes |
| 33 | PC | pyruvate carboxylase [Source:HGNC Symbol;Acc:HGNC:8636] | 2629 | 0.287 | 0.2164 | Yes |
| 34 | NARS2 | "asparaginyl-tRNA synthetase 2, mitochondrial [Source:HGNC Symbol;Acc:HGNC:26274]" | 2631 | 0.287 | 0.2235 | Yes |
| 35 | LIG4 | DNA ligase 4 [Source:HGNC Symbol;Acc:HGNC:6601] | 2668 | 0.283 | 0.2278 | Yes |
| 36 | ATP5F1D | ATP synthase F1 subunit delta [Source:HGNC Symbol;Acc:HGNC:837] | 2701 | 0.279 | 0.2323 | Yes |
| 37 | ATP6V0C | ATPase H+ transporting V0 subunit c [Source:HGNC Symbol;Acc:HGNC:855] | 2790 | 0.271 | 0.2322 | Yes |
| 38 | LIG3 | DNA ligase 3 [Source:HGNC Symbol;Acc:HGNC:6600] | 2898 | 0.261 | 0.2304 | Yes |
| 39 | ATP5MG | ATP synthase membrane subunit g [Source:HGNC Symbol;Acc:HGNC:14247] | 2941 | 0.256 | 0.2336 | Yes |
| 40 | WARS2 | "tryptophanyl tRNA synthetase 2, mitochondrial [Source:HGNC Symbol;Acc:HGNC:12730]" | 3014 | 0.248 | 0.2342 | Yes |
| 41 | ATP5F1B | ATP synthase F1 subunit beta [Source:HGNC Symbol;Acc:HGNC:830] | 3024 | 0.247 | 0.2397 | Yes |
| 42 | SUCLA2 | succinate-CoA ligase ADP-forming subunit beta [Source:HGNC Symbol;Acc:HGNC:11448] | 3228 | 0.228 | 0.2295 | No |
| 43 | SARS2 | "seryl-tRNA synthetase 2, mitochondrial [Source:HGNC Symbol;Acc:HGNC:17697]" | 3411 | 0.210 | 0.2205 | No |
| 44 | SAE1 | SUMO1 activating enzyme subunit 1 [Source:HGNC Symbol;Acc:HGNC:30660] | 3423 | 0.209 | 0.2249 | No |
| 45 | UBA6 | ubiquitin like modifier activating enzyme 6 [Source:HGNC Symbol;Acc:HGNC:25581] | 3454 | 0.206 | 0.2278 | No |
| 46 | FPGS | folylpolyglutamate synthase [Source:HGNC Symbol;Acc:HGNC:3824] | 3489 | 0.203 | 0.2302 | No |
| 47 | SLC27A5 | solute carrier family 27 member 5 [Source:HGNC Symbol;Acc:HGNC:10999] | 3525 | 0.200 | 0.2325 | No |
| 48 | ACSM2A | acyl-CoA synthetase medium chain family member 2A [Source:HGNC Symbol;Acc:HGNC:32017] | 4080 | 0.148 | 0.1928 | No |
| 49 | ATP5F1A | ATP synthase F1 subunit alpha [Source:HGNC Symbol;Acc:HGNC:823] | 4137 | 0.143 | 0.1920 | No |
| 50 | MCCC2 | methylcrotonyl-CoA carboxylase subunit 2 [Source:HGNC Symbol;Acc:HGNC:6937] | 4363 | 0.123 | 0.1775 | No |
| 51 | DARS2 | "aspartyl-tRNA synthetase 2, mitochondrial [Source:HGNC Symbol;Acc:HGNC:25538]" | 4480 | 0.115 | 0.1713 | No |
| 52 | GMPS | guanine monophosphate synthase [Source:HGNC Symbol;Acc:HGNC:4378] | 4498 | 0.113 | 0.1728 | No |
| 53 | NARS1 | asparaginyl-tRNA synthetase 1 [Source:HGNC Symbol;Acc:HGNC:7643] | 4651 | 0.100 | 0.1634 | No |
| 54 | ACSM5 | acyl-CoA synthetase medium chain family member 5 [Source:HGNC Symbol;Acc:HGNC:26060] | 4976 | 0.074 | 0.1399 | No |
| 55 | SLC27A2 | solute carrier family 27 member 2 [Source:HGNC Symbol;Acc:HGNC:10996] | 5122 | 0.062 | 0.1301 | No |
| 56 | HARS1 | histidyl-tRNA synthetase 1 [Source:HGNC Symbol;Acc:HGNC:4816] | 5196 | 0.057 | 0.1258 | No |
| 57 | UBA5 | ubiquitin like modifier activating enzyme 5 [Source:HGNC Symbol;Acc:HGNC:23230] | 5390 | 0.041 | 0.1118 | No |
| 58 | ASNSD1 | asparagine synthetase domain containing 1 [Source:HGNC Symbol;Acc:HGNC:24910] | 5409 | 0.039 | 0.1113 | No |
| 59 | RARS1 | arginyl-tRNA synthetase 1 [Source:HGNC Symbol;Acc:HGNC:9870] | 5469 | 0.035 | 0.1076 | No |
| 60 | ATG7 | autophagy related 7 [Source:HGNC Symbol;Acc:HGNC:16935] | 5488 | 0.033 | 0.1070 | No |
| 61 | LGSN | "lengsin, lens protein with glutamine synthetase domain [Source:HGNC Symbol;Acc:HGNC:21016]" | 5550 | 0.029 | 0.1030 | No |
| 62 | TARS2 | "threonyl-tRNA synthetase 2, mitochondrial [Source:HGNC Symbol;Acc:HGNC:30740]" | 5576 | 0.026 | 0.1017 | No |
| 63 | UBA7 | ubiquitin like modifier activating enzyme 7 [Source:HGNC Symbol;Acc:HGNC:12471] | 5651 | 0.019 | 0.0964 | No |
| 64 | AARS1 | alanyl-tRNA synthetase 1 [Source:HGNC Symbol;Acc:HGNC:20] | 5703 | 0.015 | 0.0928 | No |
| 65 | ACSBG2 | acyl-CoA synthetase bubblegum family member 2 [Source:HGNC Symbol;Acc:HGNC:24174] | 5970 | -0.002 | 0.0720 | No |
| 66 | ATP5PF | ATP synthase peripheral stalk subunit F6 [Source:HGNC Symbol;Acc:HGNC:847] | 6109 | -0.013 | 0.0615 | No |
| 67 | CAD | "carbamoyl-phosphate synthetase 2, aspartate transcarbamylase, and dihydroorotase [Source:HGNC Symbol;Acc:HGNC:1424]" | 6359 | -0.032 | 0.0428 | No |
| 68 | ATP5ME | ATP synthase membrane subunit e [Source:HGNC Symbol;Acc:HGNC:846] | 6365 | -0.032 | 0.0433 | No |
| 69 | MARS1 | methionyl-tRNA synthetase 1 [Source:HGNC Symbol;Acc:HGNC:6898] | 6400 | -0.035 | 0.0415 | No |
| 70 | ATP5F1E | ATP synthase F1 subunit epsilon [Source:HGNC Symbol;Acc:HGNC:838] | 6405 | -0.036 | 0.0421 | No |
| 71 | ACSL6 | acyl-CoA synthetase long chain family member 6 [Source:HGNC Symbol;Acc:HGNC:16496] | 6505 | -0.043 | 0.0354 | No |
| 72 | PCCA | propionyl-CoA carboxylase subunit alpha [Source:HGNC Symbol;Acc:HGNC:8653] | 6627 | -0.052 | 0.0272 | No |
| 73 | HLCS | holocarboxylase synthetase [Source:HGNC Symbol;Acc:HGNC:4976] | 6759 | -0.060 | 0.0185 | No |
| 74 | KARS1 | lysyl-tRNA synthetase 1 [Source:HGNC Symbol;Acc:HGNC:6215] | 6837 | -0.066 | 0.0141 | No |
| 75 | UBA3 | ubiquitin like modifier activating enzyme 3 [Source:HGNC Symbol;Acc:HGNC:12470] | 6936 | -0.075 | 0.0083 | No |
| 76 | LIG1 | DNA ligase 1 [Source:HGNC Symbol;Acc:HGNC:6598] | 6944 | -0.075 | 0.0096 | No |
| 77 | LARS2 | "leucyl-tRNA synthetase 2, mitochondrial [Source:HGNC Symbol;Acc:HGNC:17095]" | 7017 | -0.081 | 0.0060 | No |
| 78 | GARS1 | glycyl-tRNA synthetase 1 [Source:HGNC Symbol;Acc:HGNC:4162] | 7069 | -0.085 | 0.0042 | No |
| 79 | UBA1 | ubiquitin like modifier activating enzyme 1 [Source:HGNC Symbol;Acc:HGNC:12469] | 7266 | -0.100 | -0.0086 | No |
| 80 | MDM2 | MDM2 proto-oncogene [Source:HGNC Symbol;Acc:HGNC:6973] | 7273 | -0.101 | -0.0066 | No |
| 81 | TTLL5 | tubulin tyrosine ligase like 5 [Source:HGNC Symbol;Acc:HGNC:19963] | 7348 | -0.106 | -0.0097 | No |
| 82 | MCCC1 | methylcrotonyl-CoA carboxylase subunit 1 [Source:HGNC Symbol;Acc:HGNC:6936] | 7401 | -0.111 | -0.0110 | No |
| 83 | MOCS3 | molybdenum cofactor synthesis 3 [Source:HGNC Symbol;Acc:HGNC:15765] | 7752 | -0.138 | -0.0349 | No |
| 84 | ACSS3 | acyl-CoA synthetase short chain family member 3 [Source:HGNC Symbol;Acc:HGNC:24723] | 7821 | -0.143 | -0.0367 | No |
| 85 | GATC | glutamyl-tRNA amidotransferase subunit C [Source:HGNC Symbol;Acc:HGNC:25068] | 7852 | -0.144 | -0.0354 | No |
| 86 | ATP6V1A | ATPase H+ transporting V1 subunit A [Source:HGNC Symbol;Acc:HGNC:851] | 8013 | -0.156 | -0.0440 | No |
| 87 | ACSL3 | acyl-CoA synthetase long chain family member 3 [Source:HGNC Symbol;Acc:HGNC:3570] | 8170 | -0.169 | -0.0520 | No |
| 88 | ACSM1 | acyl-CoA synthetase medium chain family member 1 [Source:HGNC Symbol;Acc:HGNC:18049] | 8210 | -0.172 | -0.0507 | No |
| 89 | CTPS2 | CTP synthase 2 [Source:HGNC Symbol;Acc:HGNC:2520] | 8238 | -0.174 | -0.0485 | No |
| 90 | TTLL4 | tubulin tyrosine ligase like 4 [Source:HGNC Symbol;Acc:HGNC:28976] | 8389 | -0.185 | -0.0556 | No |
| 91 | GCLC | glutamate-cysteine ligase catalytic subunit [Source:HGNC Symbol;Acc:HGNC:4311] | 8534 | -0.196 | -0.0619 | No |
| 92 | SLC27A3 | solute carrier family 27 member 3 [Source:HGNC Symbol;Acc:HGNC:10997] | 8596 | -0.201 | -0.0616 | No |
| 93 | NADSYN1 | NAD synthetase 1 [Source:HGNC Symbol;Acc:HGNC:29832] | 8858 | -0.222 | -0.0765 | No |
| 94 | CARS2 | "cysteinyl-tRNA synthetase 2, mitochondrial [Source:HGNC Symbol;Acc:HGNC:25695]" | 8958 | -0.228 | -0.0785 | No |
| 95 | CARS1 | cysteinyl-tRNA synthetase 1 [Source:HGNC Symbol;Acc:HGNC:1493] | 9062 | -0.237 | -0.0806 | No |
| 96 | ACSL5 | acyl-CoA synthetase long chain family member 5 [Source:HGNC Symbol;Acc:HGNC:16526] | 9136 | -0.243 | -0.0802 | No |
| 97 | ACACA | acetyl-CoA carboxylase alpha [Source:HGNC Symbol;Acc:HGNC:84] | 9177 | -0.246 | -0.0772 | No |
| 98 | ACSF2 | acyl-CoA synthetase family member 2 [Source:HGNC Symbol;Acc:HGNC:26101] | 9219 | -0.250 | -0.0741 | No |
| 99 | YARS1 | tyrosyl-tRNA synthetase 1 [Source:HGNC Symbol;Acc:HGNC:12840] | 9422 | -0.266 | -0.0833 | No |
| 100 | PAICS | phosphoribosylaminoimidazole carboxylase and phosphoribosylaminoimidazolesuccinocarboxamide synthase [Source:HGNC Symbol;Acc:HGNC:8587] | 9475 | -0.272 | -0.0805 | No |
| 101 | GART | "phosphoribosylglycinamide formyltransferase, phosphoribosylglycinamide synthetase, phosphoribosylaminoimidazole synthetase [Source:HGNC Symbol;Acc:HGNC:4163]" | 9861 | -0.307 | -0.1029 | No |
| 102 | LRRC47 | leucine rich repeat containing 47 [Source:HGNC Symbol;Acc:HGNC:29207] | 10062 | -0.327 | -0.1104 | No |
| 103 | IARS1 | isoleucyl-tRNA synthetase 1 [Source:HGNC Symbol;Acc:HGNC:5330] | 10154 | -0.337 | -0.1090 | No |
| 104 | AARSD1 | alanyl-tRNA synthetase domain containing 1 [Source:HGNC Symbol;Acc:HGNC:28417] | 10222 | -0.343 | -0.1057 | No |
| 105 | AACS | acetoacetyl-CoA synthetase [Source:HGNC Symbol;Acc:HGNC:21298] | 10226 | -0.344 | -0.0973 | No |
| 106 | MTHFD1 | "methylenetetrahydrofolate dehydrogenase, cyclohydrolase and formyltetrahydrofolate synthetase 1 [Source:HGNC Symbol;Acc:HGNC:7432]" | 10249 | -0.346 | -0.0903 | No |
| 107 | WARS1 | tryptophanyl-tRNA synthetase 1 [Source:HGNC Symbol;Acc:HGNC:12729] | 10289 | -0.350 | -0.0846 | No |
| 108 | YARS2 | tyrosyl-tRNA synthetase 2 [Source:HGNC Symbol;Acc:HGNC:24249] | 10299 | -0.350 | -0.0765 | No |
| 109 | UBA2 | ubiquitin like modifier activating enzyme 2 [Source:HGNC Symbol;Acc:HGNC:30661] | 10360 | -0.357 | -0.0722 | No |
| 110 | GCLM | glutamate-cysteine ligase modifier subunit [Source:HGNC Symbol;Acc:HGNC:4312] | 10439 | -0.366 | -0.0691 | No |
| 111 | CTPS1 | CTP synthase 1 [Source:HGNC Symbol;Acc:HGNC:2519] | 10569 | -0.380 | -0.0697 | No |
| 112 | ACSL1 | acyl-CoA synthetase long chain family member 1 [Source:HGNC Symbol;Acc:HGNC:3569] | 10705 | -0.394 | -0.0703 | No |
| 113 | BTRC | beta-transducin repeat containing E3 ubiquitin protein ligase [Source:HGNC Symbol;Acc:HGNC:1144] | 10836 | -0.409 | -0.0702 | No |
| 114 | TTLL12 | tubulin tyrosine ligase like 12 [Source:HGNC Symbol;Acc:HGNC:28974] | 10861 | -0.411 | -0.0618 | No |
| 115 | PFAS | phosphoribosylformylglycinamidine synthase [Source:HGNC Symbol;Acc:HGNC:8863] | 10943 | -0.424 | -0.0575 | No |
| 116 | ACACB | acetyl-CoA carboxylase beta [Source:HGNC Symbol;Acc:HGNC:85] | 10955 | -0.425 | -0.0477 | No |
| 117 | VARS1 | valyl-tRNA synthetase 1 [Source:HGNC Symbol;Acc:HGNC:12651] | 11035 | -0.435 | -0.0429 | No |
| 118 | ACSL4 | acyl-CoA synthetase long chain family member 4 [Source:HGNC Symbol;Acc:HGNC:3571] | 11327 | -0.475 | -0.0538 | No |
| 119 | TARS1 | threonyl-tRNA synthetase 1 [Source:HGNC Symbol;Acc:HGNC:11572] | 11357 | -0.480 | -0.0440 | No |
| 120 | TTLL3 | tubulin tyrosine ligase like 3 [Source:HGNC Symbol;Acc:HGNC:24483] | 11449 | -0.496 | -0.0386 | No |
| 121 | ITCH | itchy E3 ubiquitin protein ligase [Source:HGNC Symbol;Acc:HGNC:13890] | 11800 | -0.549 | -0.0523 | No |
| 122 | TRIM25 | tripartite motif containing 25 [Source:HGNC Symbol;Acc:HGNC:12932] | 11834 | -0.556 | -0.0409 | No |
| 123 | ASNS | asparagine synthetase (glutamine-hydrolyzing) [Source:HGNC Symbol;Acc:HGNC:753] | 11913 | -0.574 | -0.0325 | No |
| 124 | TTLL7 | tubulin tyrosine ligase like 7 [Source:HGNC Symbol;Acc:HGNC:26242] | 11937 | -0.580 | -0.0198 | No |
| 125 | ADSS2 | adenylosuccinate synthase 2 [Source:HGNC Symbol;Acc:HGNC:292] | 12376 | -0.701 | -0.0364 | No |
| 126 | LARS1 | leucyl-tRNA synthetase 1 [Source:HGNC Symbol;Acc:HGNC:6512] | 12415 | -0.712 | -0.0215 | No |
| 127 | DIP2A | disco interacting protein 2 homolog A [Source:HGNC Symbol;Acc:HGNC:17217] | 12433 | -0.719 | -0.0048 | No |
| 128 | GLUL | glutamate-ammonia ligase [Source:HGNC Symbol;Acc:HGNC:4341] | 12527 | -0.762 | 0.0071 | No |
| 129 | FARSA | phenylalanyl-tRNA synthetase subunit alpha [Source:HGNC Symbol;Acc:HGNC:3592] | 12714 | -0.878 | 0.0146 | No |
Table: GSEA details [plain text format]

  

Fig 2: GOMF\_LIGASE\_ACTIVITY      
 Blue-Pink O' Gram in the Space of the Analyzed GeneSet

  

Fig 3: GOMF\_LIGASE\_ACTIVITY: Random ES distribution      
 Gene set null distribution of ES for **GOMF\_LIGASE\_ACTIVITY**
